# Tyrosinase inhibition by *p*‐coumaric acid ethyl ester identified from camellia pollen

**DOI:** 10.1002/fsn3.2004

**Published:** 2020-12-11

**Authors:** Lijun Li, Yuchen Cai, Xu Sun, Xiping Du, Zedong Jiang, Hui Ni, Yuanfan Yang, Feng Chen

**Affiliations:** ^1^ College of Food and Biological Engineering Jimei University Xiamen China; ^2^ Fujian Provincial Key Laboratory of Food Microbiology and Enzyme Engineering Xiamen China; ^3^ Research Center of Food Biotechnology of Xiamen City Xiamen China; ^4^ Department of Food, Nutrition and Packaging Sciences Clemson University Clemson SC USA

**Keywords:** camellia pollen, inhibition, molecular docking, *p*‐coumaric acid ethyl ester, tyrosinase

## Abstract

A tyrosinase inhibitor was separated from camellia pollen with the aid of solvent fraction, macroporous adsorptive resin chromatography, and high‐speed countercurrent chromatography. The inhibitor was identified to be *p*‐coumaric acid ethyl ester (*p*‐CAEE) by nuclear magnetic resonance and mass spectrum. Its inhibitory activity (IC_50_ = 4.89 μg/ml) was about 10‐fold stronger than arbutin (IC_50_ = 51.54 μg/ml). The *p*‐CAEE inhibited tyrosinase in a noncompetitive model with the *K*_I_ and *K*_m_ of 1.83 μg/ml and 0.52 mM, respectively. Fluorescence spectroscopy analysis showed the *p*‐CAEE quenched an intrinsic fluorescence tyrosinase. UV‐Vis spectroscopy analysis showed the *p*‐CAEE did not interact with copper ions of the enzyme. Docking simulation implied the *p‐*CAEE induced a conformational change in the catalytic region and thus changed binding forces of L‐tyrosine. Our findings suggest that *p*‐CAEE plays an important role in inhibiting tyrosinase and provides a reference for developing pharmaceutical, cosmetic, and fruit preservation products using pollen.

## Graphical abstract

*p*‐Coumaric acid ethyl ester was first found in camellia pollen. The inhibition is reversible and dose‐dependent. *p*‐Coumaric acid ethyl ester altered the structure of tyrosinase. *p*‐Coumaric acid ethyl ester and pollen had potential applications for pharmaceutical, cosmetic, and fruit preservation. *p*‐Coumaric acid ethyl ester (*p*‐CAEE) can inhibit the activity of tyrosinase in a noncompetitive manner and can cause the transformation of tyrosinase.

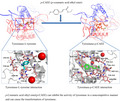

## 1 INTRODUCTION

Tyrosinase (EC 1.14.18.1) is a multifunctional copper‐containing metal enzyme with binuclear copper ions, which plays a role of speed limiting enzyme in melanin synthesis (Noh et al., 2020). In addition, tyrosinase is the main cause of fruit and vegetable browning (Yi et al., 2010). In recent years, it has been found that Parkinson's disease and melanoma are also related to tyrosinase (Carballo‐Carbajal et al., 2019). Therefore, tyrosinase inhibitors are becoming more and more valuable due to the huge markets of cosmetics, fruits preservation, and Parkinson disease prevention. For example, global industry analysts have predicted that the universal market for skin lighteners will reach $23 billion by 2020 (Pillaiyar et al., 2017).

Usually, tyrosinase inhibitors are purified from plant tissues using a combined procedure consisting of solvent extraction and chromatography separation, followed by structure identification by high‐performance chromatography, mass spectrum, and nuclear magnetic resonance. The inhibition of manners could be elucidated by kinetics studies (Saeed et al., 2017). Structural changes in a tyrosinase caused by inhibitors could be characterized using circular dichroism (CD), UV‐Visible spectral, and fluorescence techniques (Guo et al., 2018). Furthermore, X‐ray and computational techniques have been successfully applied to analyze the binding sites and molecular interactions between inhibitors and enzymes (Fujieda et al., 2020; Song et al., 2020).

At present, some compounds such as hydroquinone (HQ) (Patil et al., 2016), arbutin (Chang, 2009), kojic acid (Guo et al., 2018), azelaic acid (Sima et al., 2011), L‐ascorbic acid (Huang et al., 2013), ellagic acid (Zhu & Gao, 2008), and tranexamic acid (Hsieh et al., 2013) have been identified to inhibit tyrosinase. However, HQ can cause skin irritation (Kim et al., 2017). Arbutin and kojic acid are highly cytotoxic and unstable in oxygen and water (Fujimoto et al., 1998). L‐Ascorbic acid is heat sensitive and easy to deteriorate (Spinola et al., 2013). Ellagic acid has poor bioavailability (Arulmozhi et al., 2013) and potential adverse interactions with taxane chemotherapy (Eskra et al., 2019). So it is of interest to identify and characterize novel tyrosinase inhibitors from plants.

Pollen that plays an essential role in the sexual propagation of plants carries a variety of nutrients and bioactive compounds necessary for survival and fusion with a female gamete (Edlund et al., 2004). It contains an abundance of proteins, lipids, vitamins, minerals (Ares et al., 2018), and bioactive compounds such as phenolic compounds, flavonoids, and tocopherols (Almeida‐Muradian et al., 2005; Li, Wang et al., 2018; Li, Yuan, et al., 2018). Moreover, studies have demonstrated that pollen has the ability to inhibit tyrosinase (Zhang et al., 2015) and regulate melanogenesis of B16 cells in the cAMP/MITF/TYR pathway (Sun et al., 2017). Some tyrosinase inhibitors, including kaempferol, levulinic acid, and 5‐hydroxymethyl furfural, have been characterized from pollens (Yang et al., 2016, 2019). Thus far, tyrosinase inhibitors in pollen have not been sufficiently elucidated.

In the present study, a new tyrosinase inhibitor has been separated and characterized from camellia pollen. The specific contents included the following: (a) separate and purify the tyrosinase inhibitors by macroporous adsorption resin chromatography and HSCCC; (b) identify the structure of the tyrosinase inhibitor; (c) characterize the inhibition by kinetic, CD, fluorescence, and UV‐Visible spectral analyses; and (d) elucidate the interaction between the inhibitor and the enzyme using computational simulation. This study could help people to understand the tyrosinase inhibition activity of pollens and provide a reference for developing effective tyrosinase inhibitors for whitening agents and other beneficial products.

## 2 MATERIALS AND METHODS

### 2.1 Chemicals and reagents

Analytical‐grade ethanol, petroleum ether, ethyl acetate, and n‐butanol were purchased from Sinopharm Chemical Reagent Corporation. Chromatographic grade acetonitrile was purchased from Tedia Company Inc. Diaion HP‐20 macroporous adsorption resin was purchased from Beijing Green Herbs Co., Ltd. Mushroom tyrosinase (E.C.1.14.18.1), L‐tyrosine, and standards of arbutin and *p*‐coumaric acid ethyl ester were purchased from Merck Life Science Co., Ltd. Camellia pollen was purchased from Beijing Tong Ren Tang Co., Ltd.

### 2.2 Purification tyrosinase inhibitor from pollen

Dry camellia pollen (500 g) was powdered by a kitchen blender (JP‐500C, Jiu Pin Dian Qi Co., Ltd). Then, the pollen powder was extracted with 5 L 95% (v/v) ethanol for three times (each for 8 hr) at 50°C. The extracts were pooled and vacuum concentrated to yield a slurry (200 g) that was then further extracted by 1 L 99.5% (v/v) ethyl acetate for four times. The ethyl acetate extract was vacuum concentrated at 50°C. The concentrate (10 g) was dissolved in 1 L water and separated by HP‐20 macroporous adsorptive resin using batch elution by 30% (v/v) ethanol. The eluents were vacuum concentrated at 50°C using a rotary evaporator. Fraction 3 of the eluent was hydrolyzed using a previously reported method with minor modifications (Nuutila et al., 2002). Briefly, 0.1 g fraction 3 eluent was dissolved in 50 ml 95% (v/v) ethanol and then mixed with 50 ml 4 M hydrochloric acid, sealed, and incubated in a water bath at 90°C for 90 min. After cooling down to room temperature, the hydrolyzate was evaporated in a rotary evaporator at 50°C under vacuum. The dried hydrolyzate was submitted to high‐speed countercurrent chromatography separation (TBE‐300C, Shanghai Tauto Biotech Co., Ltd.) using the solvent mixture of n‐hexane, ethyl acetate, methanol, and water at the ratio of 4:6:4:6 (v/v/v/v). The flow rate was 3 ml/min, and the column temperature was kept at 25°C. The effluent from the outlet of the column was monitored with a UV detector at 280 nm. Fractions were collected according to the chromatograms.

### 2.3 High‐performance liquid chromatography analysis

Agilent 1260 HPLC system (Agilent Technologies Co.) and reverse‐phase Symmetry C18 column (150 mm × 4.6 mm i.d., 3.5 µm, Waters) were used for HPLC analysis. The mobile phase was composed of ultrapure water (mobile phase A) and acetonitrile (mobile phase B). The gradient elution program was set in the following procedure: 0–5 min, 5% acetonitrile; 5–10 min, 5%–25% acetonitrile; 10–35 min, 25%–45% acetonitrile; and 35–40 min, 45%–5% acetonitrile. The column temperature was set at 35°C, the detector wavelength was set at 280 nm, and the flow rate was 0.5 ml/min.

### 2.4 Structure identification of the tyrosinase inhibitor

The nuclear magnetic resonance spectroscopy (Bruker AVANCE III 400, Bruker BioSpin Corporation) and deuterated methanol solution (CD_3_OD) were used for analysis. Mass spectrometry data were obtained in positive ionization mode from an Agilent 6460 triple quadrupole tandem mass spectrometer (Agilent Technologies Co.) with an ESI interface. The gas temperature was 325°C, gas flow was 12 L/min, nebulizer was 45 psi, sheath gas temperature was 300°C, sheath gas flow was 12 L/min, capillary voltage was 4,000 V, and nozzle voltage was 450 V.

### 2.5 Tyrosinase inhibitory activity assay

The inhibition of tyrosinase in vitro was measured by a previously reported method with minor modifications (Rezaei et al., 2018). Different concentrations of collected samples (40 µl) were transferred to a 96‐well plate and then mixed with 80 µl 20 mM phosphate buffer (pH 6.8) and 40 µl 250 U/ml tyrosinase solution. After incubation at 25°C for 10 min in the darkness, 40 µl 0.85 mM substrate L‐tyrosine was added to the mixture. The absorbance of each well was measured using a microplate reader (FLUO star OPTIMA) at wavelength 492 nm. Arbutin was severed as a positive control. Each experiment was done in triplicate, and the tyrosinase inhibitory activity (%) was calculated as flow:

$$\text{Inhibition}\;\text{(\textbackslash{}\% )}=\left[1‐\frac{A1‐A2}{A3‐A4}\right]\times 100\%$$

where *A*1 is the absorbance of solution with active tyrosinase and sample; *A*2 is the absorbance of solution with inactivated tyrosinase and sample; *A*3 is the absorbance of solution with active tyrosinase and methanol; *A*4 is the absorbance of solution with inactivated tyrosinase and methanol.

The IC_50_ value is defined as the inhibitor concentration required to reach a 50% inhibition of tyrosinase activity and measured by the linear fitting.

### 2.6 Kinetic analysis

The enzyme activity was measured at different concentrations of peak IV (0, 1.5, 2.0, 2.5, and 3.0 μg/ml) and different concentrations of tyrosinase (0, 62.5, 125, 250, and 500 U/ml). A linear regression was created in a double reciprocal plot of the reaction rate and the concentration of the substrate. In addition, the Lineweaver–Burk plot was used to determine the type of tyrosinase inhibition according to a previously described method with modifications (Espin et al., 2000). The reaction rate was measured at different concentration of the substrate L‐tyrosine (0.1, 0.2, 0.3, 0.4, and 0.5 mM), and different concentrations of peak IV (0, 1.5, 2.0, 2.5, and 3.0 μg/ml). For the noncompetitive type inhibition, Michaelis constant *K*_m_ was calculated by the equation below:

$$\frac{1}{v}=\frac{\mathrm{Km}}{V_{\max}}\left(1+\frac{\left[I\right]}{\mathrm{Ki}}\right)\frac{1}{\left[S\right]}+\frac{1}{V_{\max}}\left(1+\frac{\left[I\right]}{\mathrm{Ki}}\right)$$

where *K*_m_ is the Michaelis constant; *V*_max_ is the maximum velocity; *V* is the reaction rate, and [*S*] is the substrate concentration.

### 2.7 Circular dichroism spectroscopy assay

The CD spectroscopy (Jasco‐810 spectrophotometer, JASCO) analysis was done according to a previously reported method (Biswas et al., 2017). Briefly, 285 μL of 1.86 mg/ml tyrosinase solution was mixed with 20 μL different concentrations of peak IV solution to reach a final molar ratio of 0:1, 1:1, or 4:1. Then, each tyrosinase inhibitor mixture was injected into a 1‐cm‐path length quartz cuvette and the CD spectrum. The detection conditions were as follows: The scanning wavelength was from 190 to 250 nm, the scanning rate was 100 nm/min, the resolution time was 0.5 s, the response time was 1 s, and the bandwidth was 2 nm. After subtracting the blank (0.5% methanol solution) signal, structure parameters (α‐helices, β‐turns, β‐sheets, random Coils) were analyzed by the CDNN program v.2.1 from Applied Photophysics, Ltd.

### 2.8 Fluorescence emission spectroscopy assay

A mixture of 50 μL tyrosinase (250 U/ml) and 925 μL 20 mM phosphate buffer (pH 6.8) was incubated with 25 μL peak IV at different concentrations of 0, 100, 300, and 500 μg/ml. The emission spectra were recorded at 20°C by using a 1‐cm‐path length quartz cuvette in a Varian Cary Eclipse fluorescence spectrometer (Varian, Inc.). The excitation wavelength was 280 nm, and the emission spectra were recorded from 300 to 480 nm. The excitation and emission slits were 5 nm, the scanning rate was 1,000 nm/min, and the resolution was 1.0 nm.

### 2.9 UV‐Vis spectra analyses

Five samples were prepared, sample 1 contained 1 mg/ml of *p*‐coumaric acid ethyl ester only; sample 2 contained 0.125 mM of copper (II) sulfate solution only; sample 3 contained 500 U/ml of tyrosinase only; sample 4 was composed of 1 mg/ml of *p*‐coumaric acid ethyl ester and 0.125 mM of copper (II) sulfate solution; and sample 5 was composed of 1 mg/ml of *p*‐coumaric acid ethyl ester and 500 U/ml of tyrosinase. The change in the absorbance and wavelength on the interaction was recorded using UV‐Vis spectrophotometer with a wave scan range from 200 to 600 nm.

### 2.10 Computational simulation analysis

The crystal structure of tyrosinase (PDB code: 2Y9X) (Chai et al., 2018) was applied as model of molecular docking. ChemBioDraw Ultra 12.0 was used to prepare the three‐dimensional (3D) structures of the ligands, such as *p‐*coumaric acid ethyl ester and L‐tyrosine. The molecular dynamic simulation procedure was performed by Discovery Studio software (Accelrys Inc.). Molecular docking analysis was performed using AutoDock 4.2 software (The Scripps Research Institute). The final conformation and pdbqt topology file were extracted for docking. Protein–ligand complexes and their residue changes were visualized using PyMOL software (DeLano Scientific LLC) and Discovery Studio software.

### 2.11 Statistical analysis

All samples were analyzed and evaluated in triplicate. The results were expressed as a mean ± *SD*, and the data were analyzed by using one‐way ANOVA followed by a *t* test to determine any significant differences. The values of *p* < .05 were considered as statistically significant.

## 3 RESULTS AND DISCUSSION

### 3.1 Purification and identification of tyrosinase inhibitor

**Figure 1:**
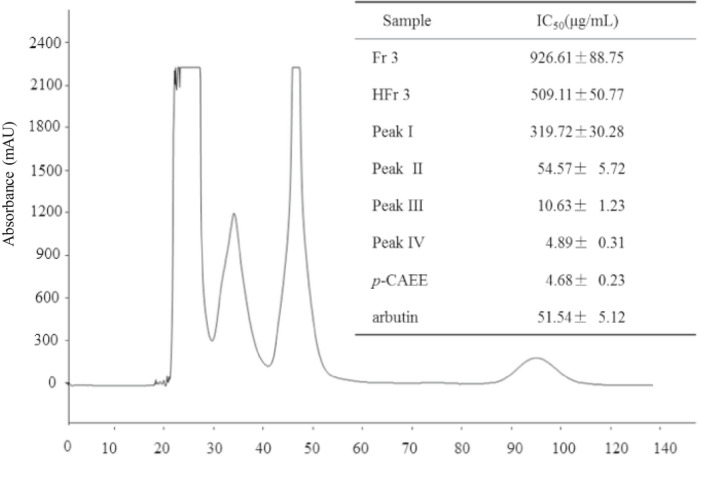
High‐speed countercurrent chromatogram and the tyrosinase inhibitory activity of the eluents. Fr3, fraction 3; HFr3, hydrochloric fraction 3; p‐CAEE, *p*‐coumaric acid ethyl ester

Figure 1 shows that Peak I, Peak II, Peak III, and Peak IV were separated from hydrochloric fraction 3. In addition, Peak IV presents a much stronger tyrosinase inhibitory activity (IC_50_ = 4.89 μg/ml) than Peak III (IC_50_ = 10.63 μg/ml), Peak II (IC_50_ = 54.57 μg/ml), Peak I (IC_50_ = 319.72 μg/ml), and the positive control arbutin (IC_50_ = 51.54 μg/ml).

**Figure 2:**
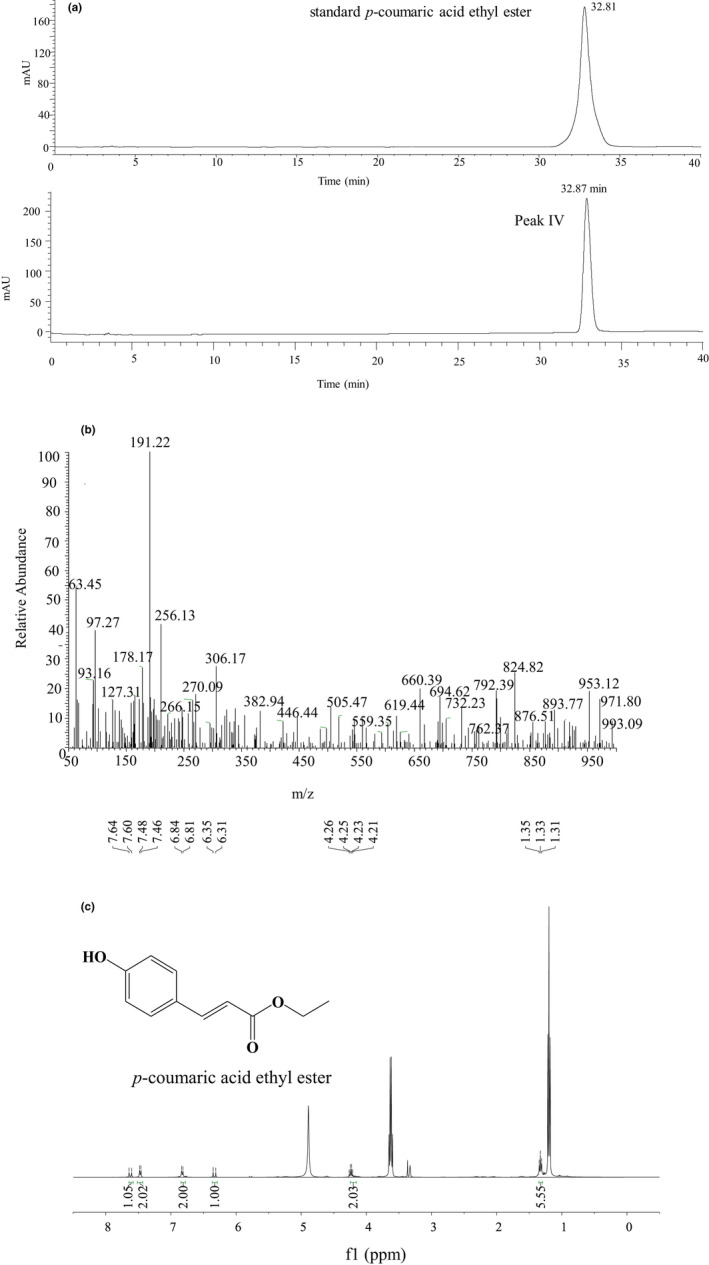
Identification analysis of peak IV. (a) High‐performance liquid chromatography. (b) Mass spectrum. (c) 1H nuclear magnetic resonance spectrum

Figure 2a shows the HPLC analysis of Peak IV and standard *p*‐coumaric acid ethyl ester. The retention time of Peak IV was 32.87 min, and standard *p*‐coumaric acid ethyl ester was 32.81 min. Figure 2b shows the quasi molecular ion peaks of peak IV was 191.22 m/z [M‐H] ^‐^, indicating the molecular weight of 192 Dalton. Previous study reports that *p*‐coumaric acid ethyl ester has the same molecular weight (Mussatto et al., 2006). Figure 2c shows the nuclear magnetic resonance analysis of peak IV. ^1^H NMR (400 MHz, CD_3_OD, δ, ppm, J/Hz) of peak IV:1.35 (3H, t, J = 8, CH_3_), 4.26 (2H, q, J = 4, 8, CH_2_), 6. 35 (1H, d, J = 16, H‐2), 6.84 (2H, d, J = 8.5, H‐3′, 5′), 7.48 (2H, d, J = 8, H‐2′, 6′), and 7.64 (1H, d, J = 16, H‐3) were found. Previous study reports the same characteristics of *p*‐coumaric acid ethyl ester. Based on these results, the tyrosinase inhibition compound separated from peak IV was identified as *p*‐coumaric acid ethyl ester (C*_p_*_‐CAEE_ = 10μg/g).

*p*‐Coumaric acid ethyl ester exists in many kinds of traditional Chinese medicine such as *Spiranthes sinensis* and *Aristolochia maurrorum* (Al‐Barham et al., 2017; Lin et al., 2019). *p*‐Coumaric acid ethyl ester has the potential to be used as a treatment for glaucoma by inhibiting carbonic anhydrases (Carta et al., 2013; Supuran, 2020). It also could inhibit the in vitro mycelial growth and spore germination of *A. alternata*, reduced black spot rot in jujube fruit caused by the pathogen (Li, Wang et al., 2018; Li, Yuan, et al., 2018). *p*‐Coumaric acid ethyl ester has important anti‐inflammatory and antioxidation activities without causing gastric injury (Melo Lima Filho et al., 2019). So, *p*‐coumaric acid ethyl ester is a bioactive compound with great potential to develop medicines. In the present study, although *p*‐coumaric acid, found in propolis from different geographic origins and bee pollen, has been shown to be a competitive inhibitor of tyrosinase that catalyzes key reactions in the melanin biosynthetic pathway (Boo, 2019; Kai et al., 2018), it was the first study showing the tyrosinase inhibition of pollen was related to *p*‐coumaric acid ethyl ester. And *p*‐coumaric acid ethyl ester exhibited strong inhibitory activities than a new series of phenylcoumarin derivatives with different numbers of hydroxyl or ether groups and bromo substituent in the scaffold (Matos et al., 2011).

### 3.2 Tyrosinase inhibitory kinetics

**Figure 3:**
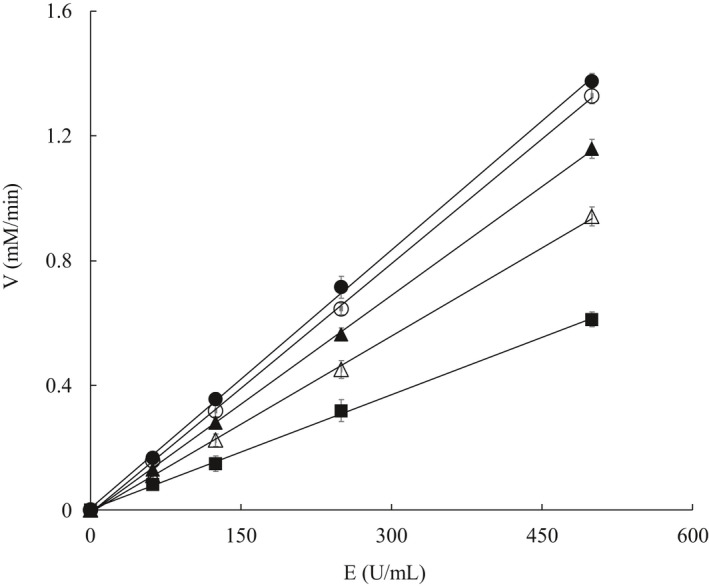
Reaction rates of tyrosinase in the presence of *p*‐coumaric acid ethyl ester with different concentrations. ●: *p*‐coumaric acid ethyl ester concentration 0 μg/ml; ○: *p*‐coumaric acid ethyl ester concentration 0.3 μg/ml; ▲: *p*‐coumaric acid ethyl ester concentration 0.4 μg/ml; △: *p*‐coumaric acid ethyl ester concentration 0.5 μg/ml; ■: *p*‐coumaric acid ethyl ester concentration 0.6 μg/ml

Figure 3 shows the relationship between tyrosinase concentration and the reaction velocity. All of the resultant plots passed through the origin point, whereas the slope decreased with the increase in *p*‐CAEE concentration. The trend is consistent with the characteristics of reversible inhibitors (Kubo et al., 2003). These results indicate that the inhibition of the *p*‐CAEE on tyrosinase was reversible by noncovalent bonds. The enzyme can be reactivated by physical removal of the bonds or inhibitors (Chen & Kubo, 2002).

**Figure 4:**
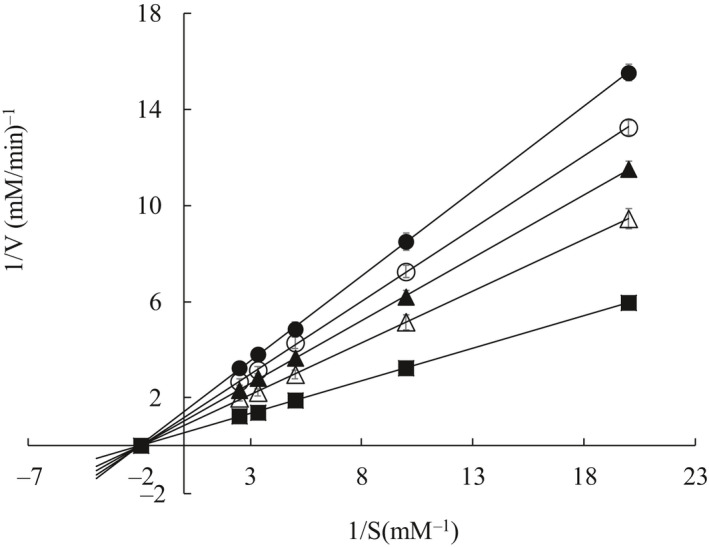
Double reciprocal plot of *p*‐coumaric acid ethyl ester on tyrosinase. ●: *p*‐coumaric acid ethyl ester concentration 0.6 μg/ml; ○: *p*‐coumaric acid ethyl ester concentration 0.5 μg/ml; ▲: *p*‐coumaric acid ethyl ester concentration 0.4 μg/ml; △: *p*‐coumaric acid ethyl ester concentration 0.3 μg/ml; ■: *p*‐coumaric acid ethyl ester concentration 0 μg/ml

Figure 4 shows that the x‐intercept (−1/*K*_m_) remained the same value but the y‐intercept (1/*V*_max_) increased with increasing concentrations of *p*‐CAEE. *K*_m_ remained constant about 0.52 mM, but *V*_max_ decreased after the addition of *p*‐CAEE. Furthermore, the parameter *K*_i_ was determined to be 1.83 μg/ml. The fixed *K*_m_ value indicates that no competition existed between the substrate and *p*‐CAEE. Early study reveals that the inhibitor binds reversibly to the free enzyme or enzyme‐substrate complex equally in case of the noncompetitive mechanism (Lopes et al., 2018). Noncompetitive inhibition means that the inhibition by inhibitors cannot be overcome by increasing substrate concentrations (Chen et al., 2005). Thus, our results indicate that *p*‐CAEE inhibited tyrosinase in a noncompetitive mechanism.

### 3.3 Circular dichroism, fluorescence, and UV‐Vis spectra analyses of the conformation changes of the tyrosinase

**Figure 5:**
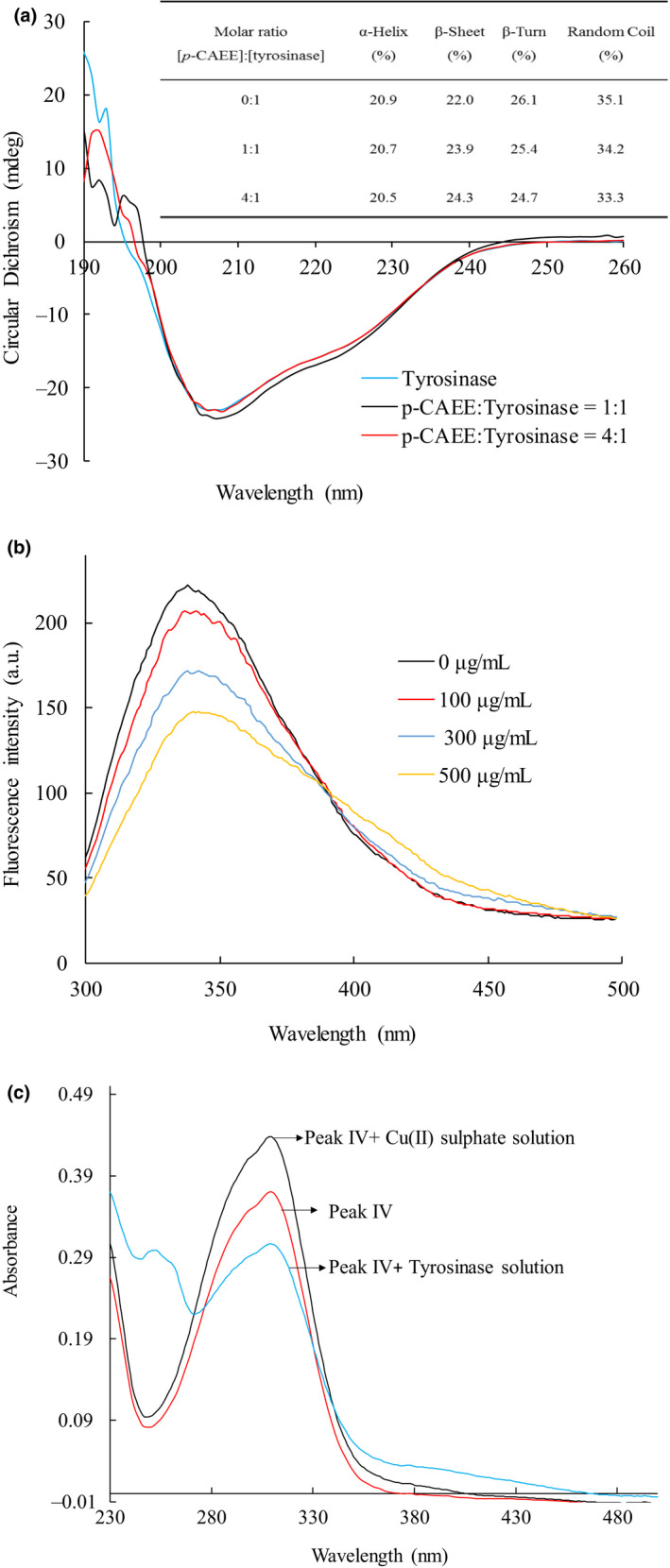
Circular dichroism, fluorescence and UV‐Vis spectra analyses. (a) Circular dichroism analyze. (b) Fluorescence analyze. (c) UV‐Vis analyze of tyrosinase in the absence and presence of *p*‐coumaric acid ethyl ester

Figure 5a shows that the spectral datum of tyrosinase exhibits 2 negative bands at 208 and 220 nm. This character is the typical feature of α‐helix resulting from the transition of amide groups in tyrosinase (VanGelder et al., 1997). Furthermore, the secondary conformation of tyrosinase was modified between 190 and 260 nm. The inserted table of Figure 5a shows that the content of α‐helix, β‐turn, and random coil decreased from 20.9, 26.1, and 35.1% to 20.5, 24.7, and 33.3% and the content of β‐sheet increased from 22.0% to 24.3% after the addition of *p*‐CAEE. This result suggests that *p*‐CAEE changed the conformation of tyrosinase.

Figure 5b shows the fluorescence spectrum of tyrosinase presented a strong emission with maximum wavelength at 340 nm. With the addition of *p*‐CAEE to the tyrosinase solution, the fluorescence intensity reduced progressively. According to a previous study, the microenvironment of tryptophan influences the fluorescence intensity at fluorescence emission maximum wavelength (340 nm) (Yousefi et al., 2013). Therefore, our result indicates that *p*‐CAEE reduced the fluorescence intensity without changing the microenvironment of tyrosinase.

Figure 5c shows sample 1 (Peak IV), sample 4 (Peak IV + Cu(II) sulfate solution), and sample 5 (Peak IV + Tyrosinase solution) had the maximum absorbance at 290 nm. Sample 2 (Cu(II) sulfate solution) and sample 3 (Tyrosinase solution) had no absorbance within the detected wavelength range. Tyrosinase is a polyphenol oxidase containing copper and 391 amino acids (Rolff et al., 2011). Some inhibitors inhibit tyrosinase by chelating the copper ion in the active site (Hridya et al., 2015) such as kojic acid (Chang, 2009) and oxalic acid (Son et al., 2000). Our results indicate that *p*‐CAEE did not directly chelate copper ions at the active site of tyrosinase.

### 3.4 Computational simulation of the molecular interaction between *p*‐coumaric acid ethyl ester and tyrosinase

**Figure 6:**
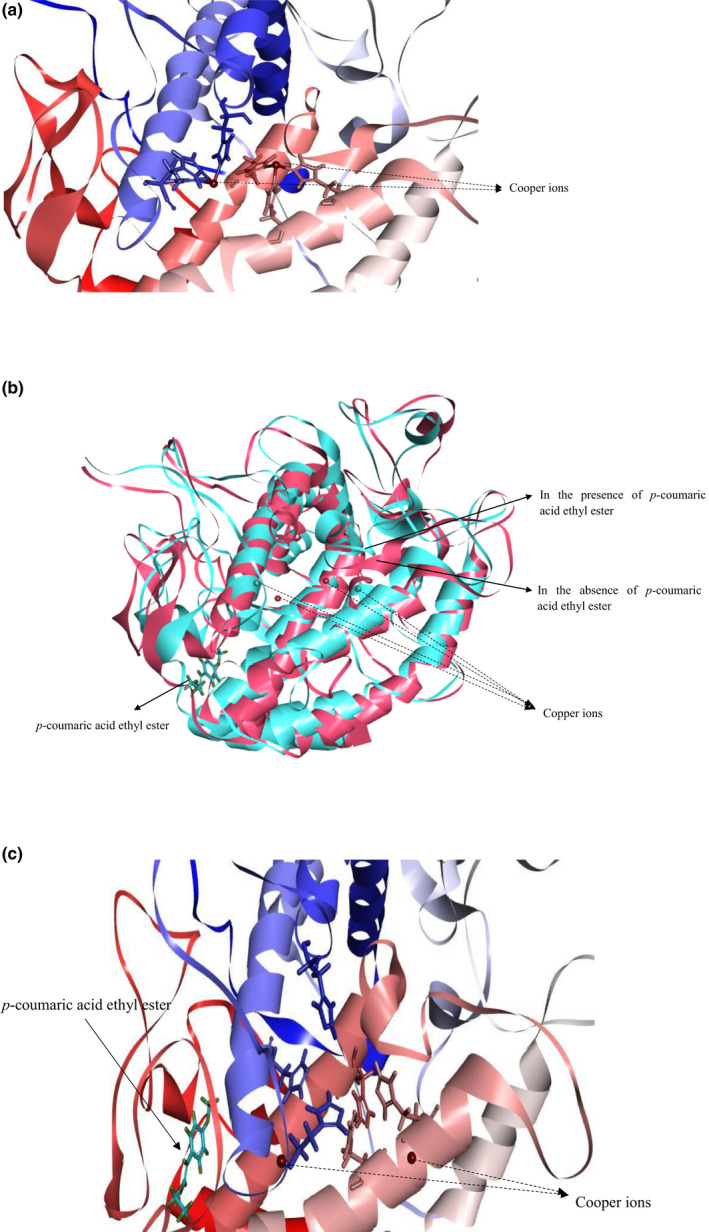
Computational docking simulation of binding between tyrosinase and *p*‐coumaric acid ethyl ester. (a) The active site of tyrosinase. (b) Superimposition of tyrosinase in the absence (red) and presence (blue) of *p*‐coumaric acid ethyl ester. (c) The active site structure of tyrosinase in the presence of *p*‐coumaric acid ethyl ester

Figure 6a shows the active site located at the bottom of the cavity on the surface. The catalytic center was constituted by two sets of three histidine residues (His 61, His 85 and His 94 of the set A, and His 259, His 263, and His 294 of the set B). Early study reports that each set is oriented by coordinately binding with copper ion (Guo et al., 2018). These copper ions are directly involved in enzymatic oxidation (Rolff et al., 2011).

Figure 6b shows that *p*‐CAEE is bound with tyrosinase at a site outside the active center and far away from the copper ions. *p*‐CAEE did not directly act on the copper ions or compete with the ligand. But *p*‐CAEE changed the location of the copper ions and the conformation of a loop adjacent to the active center.

Figure 6c shows that *p*‐CAEE did not interact with copper ions directly, but three histidine residues combining with copper ions were dispersed. The result indicates that *p*‐CAEE changed the location of copper ions in the active center.

**Figure 7:**
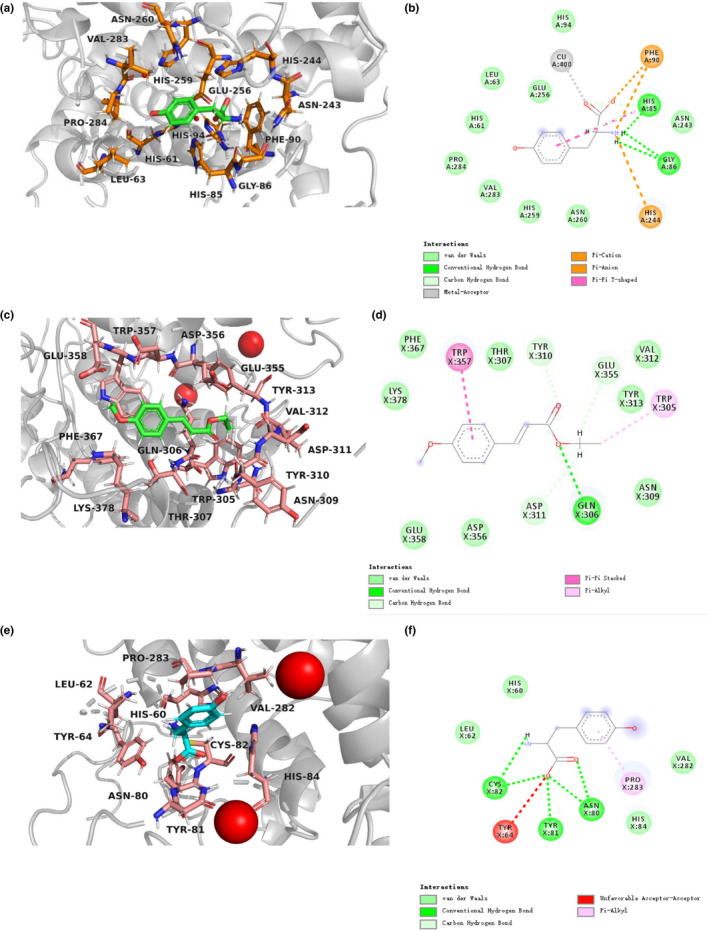
3D and 2D structural simulation of *p*‐coumaric acid ethyl ester and L‐tyrosine interacting with tyrosinase

Figure 7a,b shows the interaction between substrate L‐tyrosine and tyrosinase. L‐tyrosine bonded with His 85 and Gly 86 via hydrogen bonds, then bonded with Val 283, His 259, His 244, Glu 256, Pro 284, Asn 243, His 94, Phe 90, His 61, Leu 63, His 85, and Gly 86 via Van der Waals forces, furthermore, bonded with Cu 400 via metal interactions, last bonded with Phe 90 and His 244 through hydrophobic interactions.

Figure 7c,d shows the interaction between inhibitor *p*‐CAEE and tyrosinase. *p*‐CAEE bonded with tyrosinase via Phe 367, Lys 378, Thr 307, Val 312, Tys 313, Asn 309, Asp 356, and Glu 358 through Van der Waals forces, then bonded with Trp 357 and Trp 305 through hydrophobic interactions, furthermore bonded with Tyr 310, Glu 355 and Asp 311 by carbon‐hydrogen bonds, last bonded with Gln306 through hydrophobic interactions.

Figure 7d,e shows the interaction between substrate L‐tyrosine and tyrosinase inhibitor *p*‐CAEE complex system. L‐Tyrosine bonded with His 60, Leu 62, Val 282, and His 84 via Van der Waals forces, bonded with Tyr 64 via unfavorable acceptor‐acceptor interaction, and bound Pro 283 through hydrophobic interactions. Although the binding site of *p*‐CAEE and tyrosinase was far away from the active site, it still caused a great transformation of tyrosinase. *p*‐CAEE can make six histidines that play important roles in the catalytic activity of tyrosinase lose the chelation with copper ion, and greatly changed the binding mode of enzyme and substrate, thus produced stronger inhibition on tyrosinase than arbutin.

## 4 CONCLUSION

*p*‐CAEE found in pollen could inhibit tyrosinase. Kinetic studies elucidated that *p*‐coumaric acid ethyl ester noncompetitively inhibited the tyrosinase. The *p*‐CAEE changed the secondary conformation of tyrosinase significantly. The *p*‐CAEE had a quenching effect on the intrinsic fluorescence of tyrosinase. The *p*‐CAEE did not chelate the copper ions of the enzyme and induced a conformational change in the catalytic region and changed the binding forces of L‐tyrosine. This study demonstrated that the tyrosinase inhibition of Camellia pollen is related to *p*‐CAEE, providing a theoretical reference to utilize pollen in the pharmaceutical, cosmetic, and fruit preservation.

## CONFLICTS OF INTEREST

The authors declare that there is no conflict of interest regarding the publication of this paper.

## Data Availability

Data sharing is not applicable to this article as no new data were created or analyzed in this study.
